# Unlocking the Potential of Microwave Sterilization Technology in Ready-to-Eat Imitation Crab Meat Production

**DOI:** 10.3390/foods12244412

**Published:** 2023-12-07

**Authors:** Qianqian Xue, Changhu Xue, Donglei Luan, Yajing Wang, Yunqi Wen, Shijie Bi, Lili Xu, Xiaoming Jiang

**Affiliations:** 1 Food Science & Human Health Laboratory, College of Food Science and Engineering, Ocean University of China, Qingdao 266003, China; xue851668452@163.com (Q.X.); wenyq0715@163.com (Y.W.); jxm@ouc.edu.cn (X.J.); 2 Qingdao Institute of Marine Bioresources for Nutrition & Health Innovation, Qingdao 266109, China; 3 College of Food Science and Technology, Shanghai Ocean University, Shanghai 201306, China; wangyayajing@163.com; 4 College of Food Science and Pharmacy, Xinjiang Agricultural University, Urumqi 830052, China; bsj19941001@163.com; 5 Institute of Agro-Food Science and Technology, Shandong Academy of Agricultural Sciences, Jinan 250100, China; xulili8339@163.com

**Keywords:** surimi products, microwave technology, retort sterilization, quality traits, processing time

## Abstract

Microwave sterilization is a novel potential sterilization technology to improve food quality. An industrial microwave sterilization system was used to sterilize imitation crab meat under thermal processing intensity F_0_ = 1, 2, 3. The characteristics of the microwave process, such as heating rate, processing time, and C_100_, were calculated. In addition, the quality of processed imitation crab meat was investigated. Compared with the conventional retort method, microwave sterilization significantly shortened the processing time of imitation crab meat by 63.71% to 72.45%. Under the same thermal processing intensity, microwave sterilization has demonstrated better results than retort sterilization in terms of water-holding capacity, color, and texture. Furthermore, microwave-treated imitation crab meat ingredients had a greater capacity to bind water molecules and obtained a more appropriate secondary protein structure. In addition, microwave technology can better preserve the unsaturated fatty acids (UFA) of imitation crab meat, which are 9.14%, 1.19%, and 0.32% higher than the traditional method at F_0_ = 1, 2, 3. The results would provide useful data for the subsequent research and development of ready-to-eat surimi products.

## 1. Introduction

Surimi products are myofibrillar protein products obtained from deboned fish meat after cleaning and dehydration, which have the characteristics of low cost and high value [1,2]. The surimi market has substantial potential for development, as the surimi product output in China reached 2.76 million tonnes in 2021, which represents a 6.47% increase from the previous year [3]. Imitation crab meat is a product made from surimi, which is widely known for resembling the appearance and texture of snow crab. Nevertheless, the processing of imitation crab meat into ready-to-eat foods has resulted in several quality degradation phenomena, such as darkening of color, loss of springiness, and sticking of fibrous texture [4,5]. The conventional heat transfer process of solid food is primarily based on heat conduction, which takes a long time to transfer heat from the heating medium to the imitation crab meat [6]. Long-time heat treatment leads to a reduction in the physical and nutritional quality of solid food and an increase in energy consumption [7]. Concerns about food quality and energy have led the food industry to seek new technologies that meet conventional sterilization goals while overcoming associated drawbacks.

In this instance, emerging sterilization technologies such as ohmic heating, microwave heating, irradiation, pulsed electric field, high hydrostatic pressure, and their combinations are gradually being investigated as alternatives to conventional methods [8]. Microwave heating is an innovative technology that converts electrical energy into thermal energy by the rotation of water molecules and ions in an alternating field [9]. Microwave heating has the advantages of shortening heating time, improving efficiency, and reducing food quality damage owing to the high penetration of microwaves [10,11]. Microwave cavities are mainly divided into multimode and single mode. It is extremely difficult to predict and control the standing wave mode in a multimode microwave cavity. The thermal pattern of food in multimode cavities is unpredictable. The single-mode cavity is able to provide a stable heating mode within a reasonable bandwidth, which is suitable for food products with varying dielectric properties [9,12]. However, there is no research on the application of single-mode microwaves to imitation crab meat to produce ready-to-eat products that can be stored at room temperature.

Our previous research has studied the sterilization time and quality of *Oncorhynchus keta* Walbaum fillets, oil-soaked saury, duck meat, and rainbow trout fillets. The result showed that microwave has the potential to reduce the sterilization time (about 48.0–57.0%), inhibit color deterioration, maintain texture characteristics, and better retain nutrients such as lipids and amino acids, compared with conventional sterilization [13,14,15,16]. Therefore, the application of a single-mode microwave-assisted hot water sterilization system to imitation crab meat may bring about some good changes, such as improving product quality and reducing sterilization time.

The objective of this study was to evaluate the feasibility of using microwave sterilization to produce ready-to-eat imitation crab meat and provide recommendations for the production of crab imitation meat. The characteristics of microwave and conventional processes (F_0_ = 1, 2, 3), such as heating rate, processing time, and C_100_, were calculated. In addition, the quality traits of sterilized imitation crab meat were studied. These results would provide experimental data for imitation crab meat quality promotion and control.

## 2. Materials and Methods

### 2.1. Sample Preparation 

Formed but unsterilized imitation crab meat was provided by Zhejiang Xingye Group Co., Ltd., (Zhoushan, China). A layer of 5 pieces of imitation crab meat (70.82 ± 0.59 g) was placed in 11 × 16 cm^2^ polyamide and cooking grade cast polypropylene package (PA + PRPP) retort bags (Shijiazhuang Xilong, Shijiazhuang, China) and sealed with a Henkelman Jumbo 35 vacuum bag sealer (Henkelman Inc., CK’s-Hertogenbosch, The Netherlands) at a vacuum of 11 KPa. Prior to sterilization, imitation crab meat was preheated in water at 35 °C for 30 min.

### 2.2. Microwave Sterilization Treatment

**Figure 1:**
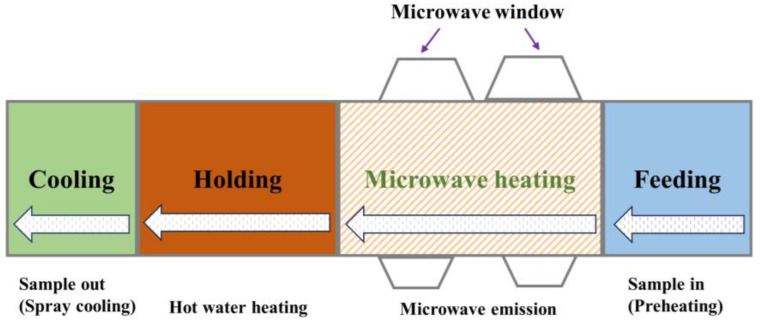
896 MHz Microwave sterilization system.

The 896 MHz single-mode microwave-assisted hot water sterilization system developed by Shanghai Ocean University was utilized for microwave sterilization (MW). The system comprises four stages: feeding, microwave heating, holding, and cooling (Figure 1) [17]. The sample enters from the feeding chamber and passes through four areas sequentially by translational motion to achieve sterilization. Cavities designed for microwave heating and holding were pumped with hot water at approximately 121 °C. The addition aims to mitigate the corner effect of microwave heating, act as a heat source for the food, and alleviate overheating in certain areas of the food. The target thermal processing intensity was achieved by adjusting the net power of the microwave, the movement speed of food, and the duration of holding.

#### 2.2.1. Heating Pattern Exploration and Determination of Cold Spot Coordinates

Because of the electric field heating characteristics of microwaves, it is necessary to explore the thermal pattern of imitation crab meat in the microwave heating system. On the basis of the research by Xue et al., the model food was prepared and cut into the size of imitation crab meat (90 × 90 × 15 mm^3^) [14]. The vacuum-packed model food was placed in the load carrier and then heated by the microwave sterilization system. Processed model food was immediately cooled to room temperature, sliced from mid-thickness, and photographed. The shooting was carried out in a studio built with a Canon camera (D850, Canon, Tokyo, Japan), and the light source in the studio was uniform and suitable. The shooting was converted to grayscale and later transformed into a pseudo-colored image with MATLAB 9.10(2021a) software. The color distribution was adjusted to improve the contrast of the image. Eventually, a suitable thermal picture was generated. The heating pattern of the sample in the microwave heating system was clearly shown in the thermal picture. The coordinates of the cold spot were determined according to the color distribution.

#### 2.2.2. Temperature Monitoring and Determination of Process Parameters

Wireless temperature sensors (PICOVACQ/1 TC, TMI Orion, Castelnau le Lez, France) were used for temperature monitoring [18]. The tip (data acquisition area) of the wireless temperature sensor was placed at the cold spot of imitation crab meat, and temperature data were acquired every 4 s. Microwave sterilization parameters under the conditions of thermal processing intensity F_0_ = 1, 2, 3 were determined by adjusting the microwave power, the food running speed, and the holding time.

Equation (1) was used to calculate the thermal processing intensity F_0_ [16]. *T*(*t*) was the temperature data (°C) collected over time for the cold spot. 121.1 °C was the reference temperature for commercial sterilization, and 10 °C was the z-value of low-acid canned indicator bacteria [2].

(1) $$F_{0}=\int_{0}^{t}10^{\frac{T(t)-121.1}{10}}dt$$

### 2.3. Conventional Retort Sterilization

Conventional retort sterilization was performed using HX-320 retort (Systec Inc., Heinsdorfergrund, Germany). Retort sterilization (RT) was a process of transferring heat from high-temperature steam to solid food, and the sterilization cold spot was located in the geometric center of imitation crab meat. Temperature monitoring and F_0_ calculation were performed in the same manner as microwave sterilization. Retort sterilization parameters under the conditions of thermal processing intensity F_0_ = 1, 2, 3 were determined by adjusting heating time.

### 2.4. Cook Value C_100_

To evaluate the cumulative thermal effect of time and temperature on food quality, the cook value C_100_ was used. The higher the cook value C_100_, the greater the reduction in food quality. The cook value C_100_ was calculated from Equation (2) [16]:

(2) $$C_{100}=\int_{0}^{t}10^{\frac{T(t)-100}{33.1}}dt$$

*T*(*t*) is the cold spot temperature as a function of time t, 100 °C is the reference temperature for food quality, and 33.1 °C is based on the average degradation of chemical components in food.

### 2.5. Quality Analysis

#### 2.5.1. Water-Holding Capacity

Water-holding capacity represents the ability of surimi products to hold water and is an important indicator of the quality evaluation of surimi products [4]. The water-holding capacity was determined according to the method of Mi [2] with appropriate modifications. The imitation crab meat sample was cut into small pieces with an average weight of 2.00 g, and their weight was recorded as m_1_(g). The small pieces were wrapped with three layers of filter paper and then transferred to a 50 mL centrifuge tube and centrifuged at 4 °C for 5 min at 8000× *g* (TGL-16M, Lu Xiangyi, Shanghai, China). The weight of the sample after centrifugation was expressed in m_2_(g), and the water-holding capacity was calculated according to the following equation [19].

(3) $$WHC\left(\%\right)=(1-\frac{m1-m2}{m1\times w})\times 100$$

#### 2.5.2. Color and White Index (WI)

A handheld colorimeter (3nh D65/10, Shenzhen Sanenshi Technology, Shenzhen, China) was used to measure the exterior and interior color parameters of imitation crab meat. Color parameters include L* (lightness), a* (redness), and b* (yellowness) values. To obtain the total color difference (DE, ΔE), the L*, a*, and b* values of the imitation crab meat exterior were plugged into Equation (4) [20].

(4) $$\Delta E={\left[{\Delta L}^{2}+\Delta a^{2}+\Delta b^{2}\right]}^{\frac{1}{2}}$$

The whiteness index (WI) is a common indicator used to assess the quality of surimi and surimi gels, and consumers generally prefer products with higher WI values. The WI value was calculated by substituting the interior color value into Equation (5) [21].

(5) $$WI=100-{\left[{\left(100-L\right)}^{2}+a^{2}+b^{2}\right]}^{\frac{1}{2}}$$

#### 2.5.3. Texture Profile Analysis (TPA)

A texture analyzer (TMS-TOUCH, Food Technology Corporation Co., Ltd., Sterling, VA, USA) was used to perform the TPA tests with a P/6 flat-bottom cylindrical probe (6 mm diameter). This test was conducted under the conditions of a 1000 N force sensing unit, 50% deformation, 1 mm/s test rate, and 0.2 N minimum starting force.

#### 2.5.4. Low-Field Nuclear Magnetic Resonance (LF-NMR)

The relaxation measurement of imitation crab meat was performed using an LF NMR analyzer (MR25, Shanghai Niumag Electronics Technology Co. Ltd., Shanghai, China). The Carr-Purcell-Meiboom-Gill (CPMG) sequence was used for the calculation of the spin-spin relaxation time (*T*_2_). The echos (NECH), time waiting (TW), and t number of scans (NS) were set to 5000, 2000 s, and 8, respectively [22]. The relaxation periods (*T*_2*b*_, *T*_21_, *T*_22_, and *T*_23_) and the relaxation signal component values (*A*_2*b*_, *A*_21_, *A*_22_, and *A*_23_) were determined. In this study, free water was denoted by the letters *T*_23_, immobile water by *T*_22_, and bound water by *T*_21_ and *T*_2*b*_.

#### 2.5.5. Fourier Transform Infrared (FTIR) Spectroscopy

The FT-IR spectra of imitation crab meat were determined using a Fourier transform infrared spectrometer (Nicolet iS10, Thermo Fisher Scientific, Waltham, MA, USA). The freeze-dried imitation crab meat powder and dried KBr were ground at a ratio of 1:100 and compressed into a light-transmitting sheet with a tableting tool [23]. The light-transmitting sheet was assessed by an infrared spectrometer with a spectral range of 500 to 4000 cm^−1^, 64 scans, and a resolution of 4 cm^−1^ in a dry room temperature environment. Omnic software (version 9.2, Thermo Fisher Scientific Corporation, Waltham, MA, USA) and Peakfit software (version 4.12, SPSS Corporation, Chicago, IL, USA) were applied to analyze the data.

#### 2.5.6. Fatty Acid Analysis

The freeze-dried imitation crab meat sample (approximately 0.30 g) was mixed with 6 mL of methanol–chloroform solution (1:2). The well-mixed solution was centrifuged at 8000 rpm for 15 min. The process was repeated three times, and the resulting supernatants were combined and concentrated under reduced pressure to obtain a lipid extract solution. Lipid extract solution (200 uL) was added to the digestion tube containing 2 mL of concentrated hydrochloric acid-methanol mixture (1:5). The digestion tube was filled with nitrogen and immersed in a water bath at 90 °C for 2 h. After the reaction was completed, 2 mL of n-hexane was added for extraction. The fatty acid composition was detected using Agilent Gas chromatography and mass spectrometry (GC–MS) equipped with HP-INNOVA capillary column (30 m × 0.32 mm × 0.25 mm), and the NIST database was used to determine the fatty acid types.

### 2.6. Statistical Analysis

All experiments were performed in triplicate at least. Experimental data were analyzed using SPSS 25 software and expressed as mean ± standard deviation. The significance of the difference was tested by one-way ANOVA (Duncan, *p* < 0.05). Graphs were generated by Matlab 9.10 (2021a) and Origin 9.8 (2021a).

## 3. Results

### 3.1. Heating Pattern and Cold Spot of Microwave Sterilization

**Figure 2:**
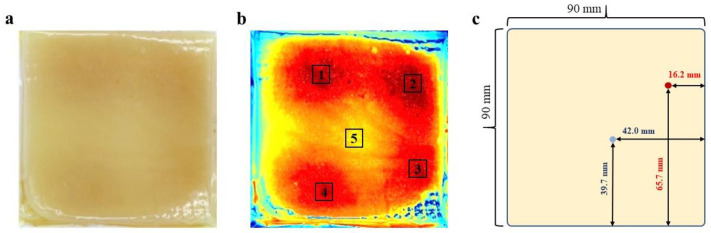
Heating pattern of model food in microwave sterilization system. Note: (**a**) Model food. (**b**) Cold and hot areas. (**c**) Coordinates of cold and hot spots.

The nonuniformity of microwave heating is a critical problem that limits its use in sterilization. The chemical marker method is an effective way to determine the cold spot of food during microwave heating [24,25]. The gellan gum model was used in the microwave heating system to obtain a clear heating pattern. The model food after microwave heating is shown in Figure 2a. The heating pattern with four hot spots and one cold spot is shown in Figure 2b. The color ranges from blue to red, with red indicating the hottest temperature and blue indicating the coldest temperature. A more detailed gridding and color comparison identified the locations of hot and cold spots in Figure 1. The coordinates originated in the lower right corner, and the length of the sides extended in a positive direction. The position of the cold spot was (42.00 mm, 39.70 mm), and the hot spot was located at (16.20 mm, 65.70 mm).

### 3.2. Heat Penetration Results and Process Parameters

**Figure 3:**
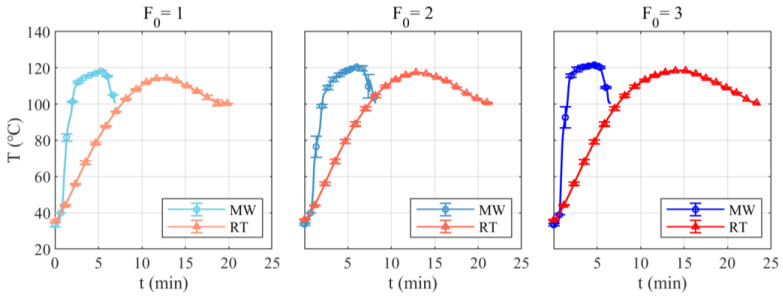
Temperature profiles at the cold spot of imitation crab meat (thermal processing intensity: F_0_ = 1, F_0_ = 2, F_0_ = 3).

The microwave and retort process parameters are shown in Table 1. Figure 3 shows the temperature change in the cold spot monitored during microwave and retort treatment. It can be seen that the microwave group has a faster heating rate and shorter processing time.

To perform quantitative statistics, the heating rate, processing time, and actual F_0_ and C_100_ of each sterilization process were calculated. The results of these calculations are shown in Table 2. The heating rate of the microwave sterilization system was 2.37–3.20 times that of the retort sterilization, resulting in a 63.71–72.45% reduction in processing time when compared with retort sterilization. There was no significant difference in the actual F_0_ between microwave and retort treatment. This suggests that the two treatment methods could be considered equivalent in terms of sterilization degree. C_100_ of microwave sterilization was significantly lower than corresponding retort sterilization, indicating that microwave-sterilized imitation crab meat had better quality.

### 3.3. Water-Holding Capacity Analysis

The results of the water-holding capacity are shown in Table 3. Water-holding capacity (WHC) is another critical quality index of surimi products. WHC depends on the interaction between the protein and water molecules of the product, reflecting the structural strength of the surimi gel product [16]. The WHC of microwave-treated imitation crab meat was higher than that of retort-treated imitation crab meat at the same thermal processing intensity (F_0_ = 1, 2, 3). The WHC of the microwave-treated imitation crab meat initially increased and then decreased, and the WHC of microwave sterilization at F_0_ = 2 was significantly higher than that of the untreated group (*p* < 0.05). The WHC of the retort-treated imitation crab meat was lower than that of the untreated imitation crab meat, and it exhibited a positive correlation with increasing F_0_ value. Treatment with retort at F_0_ = 1, 2 led to a significantly lower WHC when compared with untreated imitation crab meat (*p* < 0.05).

### 3.4. Color and White Index (WI)

Color is an important indicator that affects consumers’ appetite and purchasing desire [26]. The exterior and interior colors of the imitation crab meat before and after treatment are shown in Table 4. The external and internal color of the sterilized imitation crab meat showed a decrease in L* and an increase in a* and b*, except for the interior L* of microwave sterilization at F_0_ = 1. The use of microwave treatment on imitation crab resulted in a brighter red color, which would attract consumer interest. Both the exterior and interior color differences (ΔE) of the imitation crab meat were smaller in the microwave sterilization than in the corresponding retort sterilization. After sterilization treatment, the color of imitation crab meat in the microwave F_0_ = 1 and microwave F_0_ = 3 imitation crab meat was the closest to that of the untreated imitation crab meat.

Whiteness (WI) is one of the most important factors affecting the quality of surimi-based products [5]. The whiteness of imitation crab meat sterilized by microwaves was significantly higher than that sterilized by retort at the same thermal processing intensity (F_0_ = 1, 2, 3) (*p* < 0.05). The results indicate that microwave has significant advantages in producing foods that require a higher degree of whiteness. Therefore, microwave sterilization has a significant advantage in preserving food color. When a higher level of heat treatment is required, better food color could be obtained by adjusting the microwave power to reduce the sterilization time.

### 3.5. Texture Profile Analysis (TPA)

The TPA measurement results of the imitation crab meat before and after sterilization are shown in Table 5. The hardness of the microwave-treated crab imitation meat was similar to that of the untreated group (*p* > 0.05), but the hardness of the retort imitation crab meat was significantly higher than that of the untreated group (*p* < 0.05). The adhesiveness refers to the ability of the imitation crab meat to stick to other objects, and the adhesiveness decreased significantly after treatment (*p* < 0.05), and the decrease was greater in the retort group.

Cohesiveness represents the ability of the internal food tissues to interconnect. The cohesiveness of microwave-treated imitation crab meat was significantly lower than that of retort-treated imitation crab meat (*p* < 0.05), which might be the reason why microwave-treated imitation crab meat maintained a good fibrous texture, while retort-treated imitation crab meat appeared to be stuck together.

Springiness is the state of recovery of a deformed material after the removal of the deforming force [27]. The springiness of surimi products mainly depends on the nature of the gel network, and the degree of springiness can be used as an index to evaluate the gel strength in surimi processing [28]. Microwave-sterilized imitation crab meat had the highest springiness, which was significantly higher than untreated and retort-sterilized imitation crab meat (*p* < 0.05).

Gumminess represents the energy required to bring solid foods to a stable state during chewing [29]. Gumminess = Hardness × Cohesiveness. Chewiness is the amount of energy that is required to chew the food until it is ready to be swallowed [27]. Chewiness = Hardness × Cohesiveness × Springiness. The gumminess and chewiness of the microwave-treated imitation crab meat were significantly lower than those of the retort-treated group (*p* < 0.05).

### 3.6. LF-NMR Spin-Spin Relaxation (T_2_) Measurements

**Figure 4:**
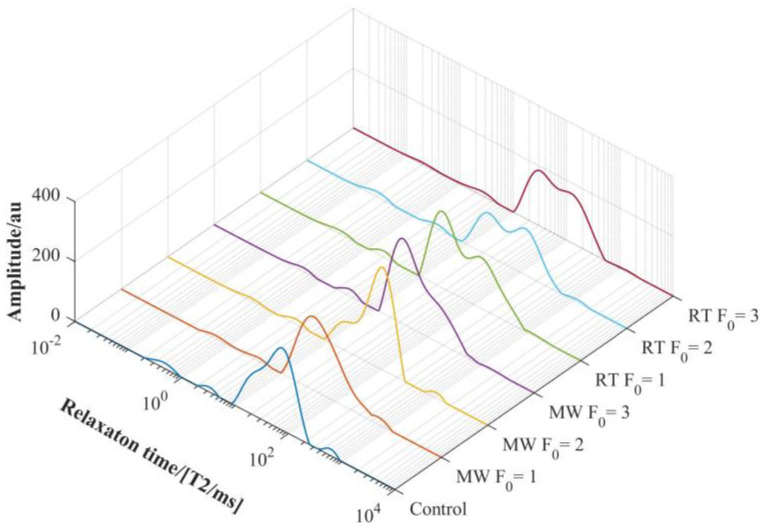
Relaxation time curves of imitation crab meat before and after heat treatment.

Low-field nuclear magnetic resonance (LF-NMR) enables rapid detection of the water mobility and distribution in food. The transverse relaxation time (*T*_2_) provides information on physicochemical changes [30]. *T*_2_ could reflect the binding force and degree of freedom for hydrogen protons, and the greater binding force for hydrogen protons can reduce the degree of freedom of hydrogen protons, and the mobility of water molecules in the sample is weakened, resulting in the shortening of *T*_2_ [31]. *T*_2_ relaxation time distribution curve of the imitation crab meat at different treatments is illustrated in Figure 4. *T*_2*b*_ (0.1–1 ms) and *T*_21_ (1–10 ms) represent the bound water in which hydrogen bonds are tightly combined with macromolecules. *T*_22_ and *T*_23_ are immobilized water ranging from 10 to 200 ms, tightly bound to a monolayer of water molecules. *T*_24_ (>200 ms) reflects free water that is poorly bound to food [32]. Four peaks were detected in untreated and microwave-treated imitation crab meat, while five were detected in retort-treated imitation crab meat.

The parameters of the relaxation time (*T*_2_) of LF-NMR are shown in Table 6. Immobilized water (*T*_22_, *T*_23_) was the most abundant component in all samples (about 92.13–94.43% of the total signal). In the composition of immobilized water, two peaks (*T*_22_, *T*_23_) appeared in the retort-treated imitation crab meat, and one peak (*T*_22_) occurred in the untreated and microwave-treated imitation crab meat, which was the reason for the change in the total number of peaks. The immobilized water of retort-treated imitation crab meat split into two peaks (*T*_22_, *T*_23_), and *T*_22_ increased with the thermal processing intensity (F_0_). Zhang et al. found that microwave treatment could inhibit the conversion of immobilized water to weaker-bound water [33]. This is consistent with the finding that the water-holding capacity of the microwave-treated imitation crab meat was significantly higher than that of the retort-sterilized (*p* < 0.05).

Researchers also found a positive correlation between water-holding capacity and LF-NMR result of low-salt *Hypophthalmichthys molitrix* surimi and Hairtail Surimi [34,35]. Measurement of water-holding capacity may also reflect the ability of water molecules to bind to food components, while LF-NMR measurement is not available.

### 3.7. Analysis of Protein Secondary Structure by Fourier Transform Infrared Spectroscopy

**Figure 5:**
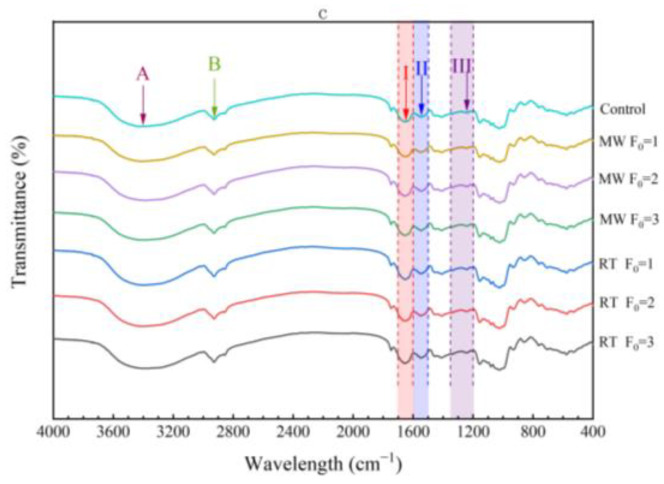
Fourier transform infrared spectra of imitation crab meat (400–4000 cm^−1^). A: amide A; B: amide B; I: amide I; II: amide II; III: amide III.

FT-IR spectroscopy is an effective means to study changes in the secondary structure of proteins and their modifications [36]. The absorbance peaks of three main bands (amide I, amide II, and amide III) and amide A and amide B bands can be seen in Figure 5. In addition, the sample has characteristic absorption peaks at around 933 cm^−1^, 1024 cm^−1^, and 1079 cm^−1^, which are caused by the stretching vibration of hydroxyl groups in antifreeze agents (sucrose, sorbitol) contained in surimi. Around 1747 cm^−1^ is an absorption peak of C=O related to lipids.

**Figure 6:**
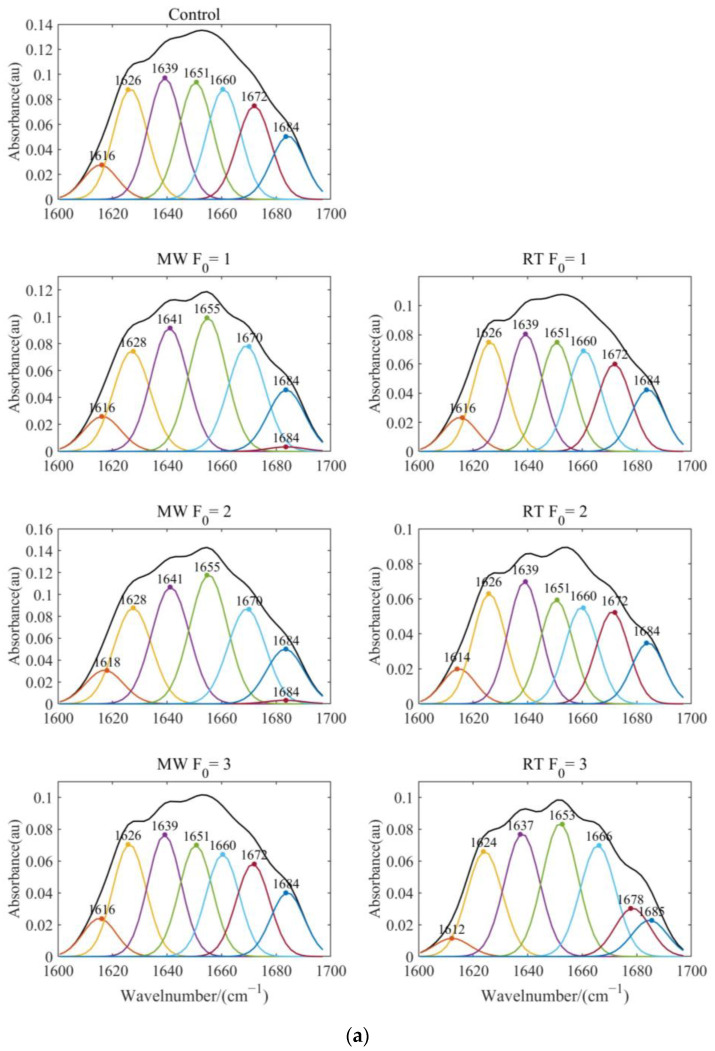

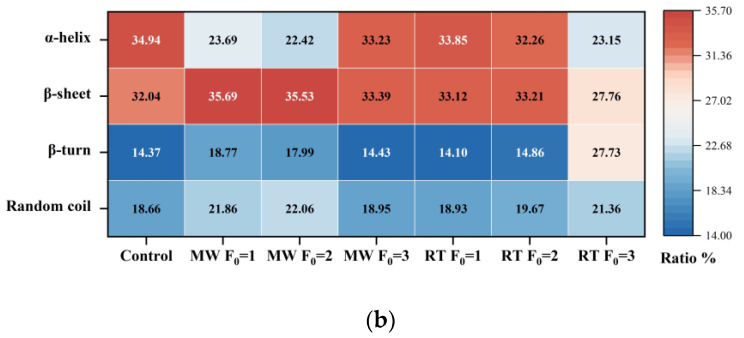
Protein secondary structure of imitation crab meat before and after sterilization. Note: (**a**) The amide region I in Fourier transform infrared (FT-IR) spectra of ICM. (**b**) Proportion of protein secondary structure.

Protein secondary structures with amide I frequency bands (1600–1700 cm^−1^) include α-helix (1645–1662), β -sheet (1613–1637; 1682–1696), β-turns (1662–1682; 1630), and unordered structures (1637–1645) [37]. Figure 6a shows the results of de-Fourier deconvolution and peak fitting of the amide I band, and Figure 6b shows the composition and content of the protein II structure. The α-helix is the main conformation of the secondary structure in natural proteins. The α-helix structure is partially unhelicated and rotated into β sheets, β-turns, or random coils when processed into surimi gel. An appropriate degree of protein unfolding and conversion of α-helices to β-sheets favors gelation [38].

Compared with retort sterilization, microwave sterilization had a higher proportion of β-sheets in the corresponding thermal processing intensity (F_0_ = 1, 2, 3). The result indicated that the imitation crab meat formed a stronger gel network after microwave sterilization [39]. Furthermore, the α-helix content of imitation crab meat was reduced, and the β-sheet content was increased at the microwave treatment of F_0_ = 1 and F_0_ = 2. The result has suggested that moderate microwave sterilization could promote the transformation of α-helices to β-sheets, which is consistent with the findings of Yan [40]. Intensifying the thermal processing intensity (F_0_ = 3) would potentially promote the regeneration of α-helices.

### 3.8. Fatty Acid Composition Analysis

As shown in Table 7, 15 types of fatty acids were present in untreated imitation crab meat, 13 kinds of fatty acids were detected in imitation crab meat with F_0_ = 1, and 12 types of fatty acids were detected in imitation crab meat with F_0_ = 2 and F_0_ = 3. C20:4 and C18:3 fatty acids were present in unprocessed imitation crab meat and disappeared after heat treatment. C18:2 fatty acids were detectable in imitation crab meat at F_0_ = 1 but were undetectable at higher degrees of heat treatment (F_0_ = 2 and F_0_ = 3). The study demonstrated that the application of sterilization treatment leads to a decrease in the quantity of fatty acids, particularly polyunsaturated fatty acids. Furthermore, the increase in the degree of sterilization directly resulted in a decline in the overall types of fatty acids present. This is consistent with Janiszewski’s findings on fatty acids in thermally processed pork [41]. Comparing the two sterilization methods, it was found that microwave sterilization could better preserve the proportion of unsaturated fatty acids. The result indicated that the degree of fat oxidation in microwave-sterilized imitation crab meat was lower. Guo et al. also found that compared with traditional sterilization methods, salmon fillets sterilized by microwave method contained less saturated fatty acids (SFA) and more unsaturated fatty acids (UFA) [17]. These results indicated that microwave sterilization of imitation crab meat could provide higher nutritional value.

## 4. Discussion

### 4.1. Processing Characteristics of Microwave Sterilization

This study proves that the high heating rate of microwave sterilization is the direct cause of short processing time. The rapid heating of the microwave for imitation crab meat is due to its high water content. Water is considered the strongest dipole, with dipole rotation and friction identified as the primary mechanisms of microwave heating [42]. Other researchers have also found that microwave sterilization reduces the processing time of Pacific whiting, surimi, and green beans [43,44]. It should be noted that microwave technology may not be universally applicable to all types of foods and requires specific analysis of factors such as the food’s dielectric properties, shape, size, and packaging materials.

Furthermore, this study demonstrates the feasibility of microwave sterilization technology by modifying microwave power, operating speed, and holding time. Regarding the flexibility of microwaves, some researchers believe that the availability of solid-state sources will further promote the applicability and accuracy of microwaves in the food industry [45]. However, it is necessary to acknowledge that the feasibility of microwaves results in an increased workload when determining process parameters.

Overall, this study is the first to investigate the heating rate and heating time of imitation crab meat using an 896 MHz microwave sterilization system, which provides a reference for other foods with similar dielectric properties.

### 4.2. Effect of Microwave Sterilization on Food Quality

In this study, microwave technology showed a high retention of physical properties and nutrients in the processing of ready-to-eat imitation crab meat. C_0_ performed well in predicting the high quality of imitation crab meat after microwave sterilization. According to some researchers, the functional and mechanical properties (such as water-holding capacity and gel strength) of surimi are improved when microwaves are used for the second heating stage [26,46,47]. The mechanism is mainly thought to be the contribution of microwaves in preventing the degradation and promoting the aggregation of myogenic fiber proteins [42]. This hypothesis was subsequently supported by several studies [48,49]. It was also observed in this study that the content of β-sheets that can improve gel properties in microwave-sterilized imitation crab meat was higher than that in retort-sterilized under the same heat treatment.

In addition, fish products have attracted the attention of researchers because they are rich in unsaturated fatty acids (UFA) [40]. However, unsaturated fatty acids are easily oxidized during thermal processing [50]. This study proves that microwave sterilization is more effective in preserving unsaturated fatty acids (UFA) and can increase their content by up to 9.14% compared with retort. Other researchers have also reported that microwave technology can reduce the degree of fatty acid oxidation [51,52,53]. It is widely accepted that the use of microwaves can reduce the exposure time of food to high temperatures, leading to the advantage [54,55]. Furthermore, this study has confirmed that as thermal processing intensity increases to F_0_ = 3, the superiority of microwave sterilization of retaining fatty acids declines. It can be seen that the difference in the ability of the microwave and retort to retain nutrients also depends on the thermal processing intensity of the food.

## 5. Conclusions

In this study, the effects of two sterilization processes (microwave and retort) on the processing characteristics and quality traits of imitation crab meat were evaluated. Microwave technology was observed to significantly shorten the processing time of imitation crab meat by 63.71–72.45%, compared with the conventional method. Microwave sterilization has demonstrated better results than retort sterilization in terms of water-holding capacity, color, and texture under the same heat treatment conditions. Furthermore, microwave-treated imitation crab meat ingredients had a greater capacity to bind water molecules and possessed a more appropriate secondary protein structure and fatty acid composition.

In microwaved sterilization, imitation crab meat exhibited better external color and interior whiteness at F_0_ = 1 and had the highest water content and water-holding capacity at F_0_ = 2. At F_0_ = 3, imitation crab meat obtained the highest springiness, while its protein secondary structure composition was inferior compared with that observed at F_0_ = 1 and F_0_ = 2. In terms of fatty acids, the imitation crab meat with microwave F_0_ = 1 had the most types of fatty acids and the highest proportion of unsaturated fatty acids. The microwave sterilization process could be used to provide better quality to meet consumer demands. In short, microwave sterilization technology demonstrates significant potential in enhancing the quality of imitation crab meat. This study has presented a novel method for the industrial manufacturing of ready-to-eat imitation crab meat.

In summary, this study provides a new technology for rapidly producing high-quality, ready-to-eat imitation crab meat. To investigate the potential impact of microwaves on the sensory quality of imitation crab meat, the volatile and nonvolatile flavor substances of imitation crab meat will be investigated in further research.

## Figures and Tables

**Table 1:** Microwave and retort process parameters of imitation crab meat.

Process	Processing	F_0_ = 1	F_0_ = 2	F_0_ = 3
Microwave	Net power of microwave (kW)	7.00	7.00	7.60
	Speed of food (cm/s)	2.00	2.00	2.50
	Microwave time (min)	1.92	1.92	1.53
	Holding time (min)	2.67	3.00	2.00
Retort	Heating temperature (°C)	121.10	121.10	121.10
	Retort time (min)	3.00	4.50	5.50

**Table 2:** Heating rate, Processing time, Actual F_0_ and C_100_ of microwave and steam sterilization.

Process	Target F_0_	Heating Rate (°C/min)	Processing Time	Actual F_0_	C_100_
Microwave	1	15.28 ± 0.4 ^Ab^	6.75 ± 0.21 ^Bb^	1.27 ± 0.06 ^Ac^	13.37 ± 0.71 ^Bb^
	2	13.50 ± 0.51 ^Ac^	7.82 ± 0.49 ^Ba^	2.46 ± 0.02 ^Ab^	17.84 ± 0.70 ^Ba^
	3	18.13 ± 0.71 ^Aa^	6.42 ± 0.12 ^Bb^	3.26 ± 0.04 ^Aa^	17.57 ± 0.35 ^Ba^
Retort	1	6.46 ± 0.14 ^Ba^	19.64 ± 0.62 ^Ac^	1.08 ± 0.08 ^Ac^	24.53 ± 1.42 ^Ac^
	2	6.27 ± 0.06 ^Ba^	21.55 ± 0.21 ^Ab^	2.26 ± 0.16 ^Ab^	32.95 ± 1.12 ^Ab^
	3	5.69 ± 0.14 ^Ba^	23.30 ± 0.52 ^Aa^	3.44 ± 0.18 ^Aa^	40.23 ± 1.65 ^Aa^

Note: The heating rate was calculated as (maximum temperature minus minimum temperature) divided by the corresponding time during sterilization. The total processing time refers to the duration between the initiation of the heating process and the cooling down to 100 °C. Capital letters indicate differences between microwave and retort processes in the same thermal processing intensity at a significance level of *p* < 0.05; lowercase letters indicate differences between thermal processing intensity F_0_ at a significance level of *p* < 0.05 (F_0_ = 1, F_0_ = 2, F_0_ = 3).

**Table 3:** Moisture content and water-holding capacity of different processed imitation crab meat.

Processing	Degree	Water-Holding Capacity (%)
Control	-	43.04 ± 3.15 ^b^
Microwave	F_0_ = 1	35.745 ± 3.48 ^d^
	F_0_ = 2	52.01 ± 0.98 ^a^
	F_0_ = 3	44.34 ± 1.61 ^b^
Retort	F_0_ = 1	34.83 ± 2.03 ^d^
	F_0_ = 2	37.98 ± 2.48 ^cd^
	F_0_ = 3	41.42 ± 2.28 ^bc^

Note: The results were mean ± standard deviation. Different lowercase letters in the same column indicate significant differences (*p* < 0.05).

**Table 4:** Exterior and interior color of imitation crab meat before and after sterilization.

	Processing		L*	a*	b*	ΔE	WI
Exterior	Control		45.73 ± 0.51 ^a^	33.10 ± 1.23 ^c^	25.20 ± 0.98 ^d^	-	-
	Microwave	F_0_ = 1	44.80 ± 0.39 ^b^	40.68 ± 0.92 ^ab^	29.18 ± 1.24 ^c^	8.66 ± 1.07 ^c^	-
		F_0_ = 2	43.28 ± 0.21 ^d^	41.78 ± 0.99 ^a^	33.20 ± 1.17 ^ab^	12.08 ± 1.26 ^a^	-
		F_0_ = 3	44.05 ± 0.52 ^c^	40.33 ± 0.38 ^b^	32.10 ± 0.73 ^b^	10.16 ± 0.18 ^bc^	-
	Retort	F_0_ = 1	41.15 ± 0.58 ^e^	40.20 ± 1.48 ^b^	32.98 ± 1.11 ^ab^	11.51 ± 1.70 ^ab^	-
		F_0_ = 2	40.30 ± 0.56 ^f^	40.73 ± 0.97 ^ab^	33.23 ± 0.99 ^ab^	12.37 ± 0.90 ^a^	-
		F_0_ = 3	40.20 ± 0.28 ^f^	41.08 ± 0.63 ^ab^	33.65 ± 0.41 ^a^	12.87 ± 0.71 ^a^	-
Interior	Control		71.48 ± 0.10 ^b^	−1.28 ± 0.17 ^d^	9.15 ± 0.47 ^b^	-	70.01 ± 0.14 ^b^
	Microwave	F_0_ = 1	72.88 ± 0.52 ^a^	−1.05 ± 0.44 ^cd^	10.83 ± 0.56 ^a^	2.23 ± 0.74 ^c^	70.77 ± 0.34 ^a^
		F_0_ = 2	69.48 ± 0.22 ^c^	−0.25 ± 0.59 ^b^	11.20 ± 0.26 ^a^	3.07 ± 0.46 ^bc^	67.48 ± 0.27 ^d^
		F_0_ = 3	70.23 ± 0.34 ^d^	−0.48 ± 0.55 ^bc^	10.90 ± 0.61 ^a^	2.36 ± 0.63 ^c^	68.28 ± 0.44 ^c^
	Retort	F_0_ = 1	68.63 ± 0.75 ^e^	0.13 ± 0.56 ^b^	10.35 ± 0.62 ^a^	3.42 ± 1.01 ^b^	66.96 ± 0.89 ^d^
		F_0_ = 2	67.33 ± 0.72 ^f^	0.15 ± 0.31 ^b^	10.63 ± 0.25 ^a^	4.65 ± 0.86 ^a^	65.64 ± 0.83 ^e^
		F_0_ = 3	66.95 ± 0.58 ^f^	0.98 ± 0.46 ^a^	10.73 ± 0.80 ^a^	5.36 ± 0.53 ^a^	65.23 ± 0.57 ^e^

Note: Different lowercase letters in the same column indicate significant differences (*p* < 0.05).

**Table 5:** TPA test results of imitation crab meat before and after treatment.

Processing		Hardness (N)	Adhesiveness (N.mm)	Cohesiveness (Ratio)	Springiness (mm)	Gumminess (N)	Chewiness (mj)
Control		7.96 ± 1.20 ^c^	0.25 ± 0.05 ^a^	0.45 ± 0.05 ^b^	4.06 ± 0.23 ^c^	3.59 ± 0.72 ^c^	14.71 ± 3.55 ^c^
Microwave	F_0_ = 1	7.26 ± 0.73 ^c^	0.10 ± 0.02 ^c^	0.40 ± 0.02 ^c^	4.78 ± 0.15 ^b^	2.93 ± 0.43 ^c^	13.97 ± 1.86 ^c^
	F_0_ = 2	8.17 ± 0.84 ^c^	0.17 ± 0.07 ^b^	0.39 ± 0.04 ^c^	4.54 ± 0.28 ^b^	3.19 ± 0.52 ^c^	14.44 ± 2.17 ^c^
	F_0_ = 3	7.17 ± 0.78 ^c^	0.14 ± 0.03 ^bc^	0.39 ± 0.05 ^c^	5.45 ± 0.34 ^a^	2.80 ± 0.63 ^c^	14.93 ± 5.59 ^c^
Retort	F_0_ = 1	11.56 ± 0.65 ^b^	0.06 ± 0.01 ^c^	0.56 ± 0.01 ^a^	3.94 ± 0.04 ^c^	6.46 ± 0.40 ^b^	25.45 ± 1.85 ^b^
	F_0_ = 2	14.64 ± 1.44 ^a^	0.06 ± 0.01 ^c^	0.55 ± 0.01 ^a^	3.90 ± 0.25 ^c^	8.03 ± 0.72 ^a^	31.36 ± 4.81 ^a^
	F_0_ = 3	13.73 ± 2.22 ^a^	0.13 ± 0.01 ^bc^	0.45 ± 0.05 ^b^	3.86 ± 0.29 ^c^	6.28 ± 1.71 ^b^	24.38 ± 7.35 ^b^

Note: Different lowercase letters in the same column indicate significant differences (*p* < 0.05).

**Table 6:** Relaxation times and proportions of peak areas of microwave and retort treatment.

Processing		*T*_2*b*_ (ms)	*T*_21_ (ms)	*T*_22_ (ms)	*T*_23_ (ms)	*T*_24_ (ms)
Control		0.43 ± 0.04 ^a^	2.88 ± 0.56 ^a^	75.65 ± 0.01 ^b^	-	613.59 ± 0.01 ^f^
Microwave	F_0_ = 1	0.52 ± 0.08 ^a^	3.31 ± 0.65 ^a^	40.37 ± 0.01 ^c^	-	657.93 ± 0.01 ^e^
	F_0_ = 2	0.35 ± 0.03 ^a^	2.48 ± 0.01 ^a^	107.23 ± 0.01 ^a^	-	1072.27 ± 0.01 ^d^
	F_0_ = 3	0.37 ± 0.13 ^a^	3.28 ± 0.32 ^a^	35.11 ± 0.01 ^d^	-	1519.91 ± 0.01 ^c^
Retort	F_0_ = 1	0.42 ± 0.16 ^a^	2.32 ± 0.23 ^a^	25.67 ± 1.27 ^f^	136.97 ± 6.76 ^a^	1149.76 ± 0.01 ^c^
	F_0_ = 2	0.27 ± 0.08 ^a^	2.85 ± 0.01 ^a^	26.56 ± 0.01 ^f^	123.28 ± 0.01 ^b^	1149.76 ± 0.01 ^b^
	F_0_ = 3	0.26 ± 0.09 ^a^	2.97 ± 0.44 ^a^	32.75 ± 0.01 ^e^	114.98 ± 0.01 ^b^	1321.94 ± 0.01 ^a^
		*A*_2*b*_ (%)	*A*_21_ (%)	*A*_22_ (%)	*A*_23_ (%)	*A*_24_ (%)
Control		2.84 ± 0.53 ^ab^	1.91 ± 0.23 ^ab^	93.16 ± 0.73 ^a^	-	2.09 ± 0.43 ^ab^
Microwave	F_0_ = 1	1.76 ± 0.45 ^b^	2.29 ± 0.37 ^ab^	93.63 ± 0.60 ^a^	-	2.32 ± 0.52 ^a^
	F_0_ = 2	2.08 ± 0.39 ^ab^	2.38 ± 0.04 ^ab^	94.36 ± 0.55 ^a^	-	1.18 ± 0.97 ^abc^
	F_0_ = 3	2.41 ± 0.44 ^ab^	2.80 ± 0.08 ^ab^	94.16 ± 0.49 ^a^	-	0.63 ± 0.02 ^c^
Retort	F_0_ = 1	3.07 ± 1.25 ^ab^	1.69 ± 0.94 ^b^	53.16 ± 1.11 ^b^	41.27 ± 0.74 ^b^	0.78 ± 0.07 ^c^
	F_0_ = 2	3.95 ± 0.56 ^a^	2.49 ± 0.22 ^ab^	40.93 ± 0.56 ^c^	51.20 ± 1.44 ^a^	1.42 ± 0.09 ^abc^
	F_0_ = 3	2.26 ± 1.04 ^ab^	2.95 ± 0.34 ^a^	52.22 ± 0.98 ^b^	41.54 ± 2.20 ^b^	1.02 ± 0.52 ^bc^

Note: Different lowercase letters in the same column indicate significant differences (*p* < 0.05).

**Table 7:** Fatty acid composition of imitation crab meat before and after treatment.

No.	Fatty Acid	Control	F_0_ = 1		F_0_ = 2		F_0_ = 3	
			MW	RT	MW	RT	MW	RT
1	C16:0	22.28 ± 5.30 ^a^	23.81 ± 0.64 ^a^	29.56 ± 2.66 ^a^	28.74 ± 1.85 ^a^	30.02 ± 2.57 ^a^	28.26 ± 0.57 ^a^	30.05 ± 1.90 ^a^
2	C17:0	1.04 ± 0.15 ^a^	1.02 ± 0.10 ^a^	1.45 ± 0.04 ^a^	1.12 ± 0.65 ^a^	1.43 ± 0.22 ^a^	1.81 ± 0.36 ^a^	1.38 ± 0.16 ^a^
3	C18:0	11.48 ± 1.01 ^ab^	10.18 ± 0.06 ^b^	12.79 ± 1.15 ^ab^	13.25 ± 1.19 ^ab^	12.56 ± 1.06 ^ab^	14.45 ± 2.57 ^a^	13.42 ± 0.90 ^ab^
4	C20:0	0.47 ± 0.04 ^ab^	0.36 ± 0.04 ^c^	0.53 ± 0.06 ^ab^	0.44 ± 0.02 ^bc^	0.57 ± 0.01 ^a^	0.54 ± 0.08 ^ab^	0.52 ± 0.05 ^ab^
5	C22:0	0.38 ± 0.05 ^bc^	0.27 ± 0.05 ^d^	0.45 ± 0.05 ^ab^	0.33 ± 0.01 ^cd^	0.49 ± 0.01 ^a^	0.42 ± 0.08 ^abc^	0.43 ± 0.04 ^abc^
	**SFA**	35.65	35.65	44.79	43.88	45.07	45.48	45.80
6	C16:1	1.38 ± 0.27 ^a^	1.40 ± 0.50 ^a^	1.37 ± 0.39 ^a^	1.10 ± 0.32 ^a^	1.60 ± 0.76 ^a^	1.11 ± 0.41 ^a^	1.41 ± 0.58 ^a^
7	C20:1	0.67 ± 0.09 ^abc^	0.57 ± 0.05 ^bc^	0.74 ± 0.04 ^ab^	0.49 ± 0.21 ^c^	0.89 ± 0.04 ^a^	0.75 ± 0.09 ^ab^	0.78 ± 0.06 ^ab^
8	C18:1	0.26 ± 0.01 ^b^	0.18 ± 0.11 ^b^	0.38 ± 0.04 ^b^	48.61 ± 2.60 ^a^	45.11 ± 4.73 ^a^	45.34 ± 4.33 ^a^	45.47 ± 3.79 ^a^
9	C22:1	1.37 ± 0.36 ^b^	1.36 ± 0.41 ^b^	2.17 ± 0.32 ^ab^	2.41 ± 0.22 ^ab^	2.3 ± 0.38 ^ab^	3.06 ± 0.86 ^a^	2.28 ± 0.08 ^ab^
	**MUFA**	3.68	3.52	4.67	52.60	49.90	50.26	49.94
10	C20:4	0.36 ± 0.10	-	-	-	-	-	-
11	C20:5	1.11 ± 0.34 ^ab^	1.01 ± 0.18 ^ab^	1.23 ± 0.14 ^ab^	0.90 ± 0.21 ^b^	1.46 ± 0.11 ^a^	1.22 ± 0.15 ^ab^	1.22 ± 0.12 ^ab^
12	C18:2	55.90 ± 6.67 ^a^	57.25 ± 1.12 ^a^	46.21 ± 4.28 ^a^	-	-	-	-
13	C18:3	0.20 ± 0.07	-	-	-	-	-	-
14	C22:6	2.92 ± 0.95 ^a^	2.41 ± 0.4 ^a^	2.88 ± 0.41 ^a^	2.47 ± 0.63 ^a^	3.30 ± 0.19 ^a^	2.84 ± 0.36 ^a^	2.83 ± 0.28 ^a^
15	C22:5	0.18 ± 0.07 ^a^	0.15 ± 0.03 ^a^	0.22 ± 0.03 ^a^	0.15 ± 0.05 ^a^	0.26 ± 0.01 ^a^	0.2 ± 0.03 ^a^	0.21 ± 0.03 ^a^
	**PUFA**	60.67	60.83	50.54	3.52	5.02	4.26	4.26
	**UFA**	64.35	64.35	55.21	56.12	54.93	54.52	54.20

Note: Different lowercase letters in the same row indicate significant differences (*p* < 0.05).

## Data Availability

Data is contained within the article.
